# Supplementary material for: Mapping the evolution of fertility support policies in China: A content and instrumental analysis
Source: PLoS One. 2025 Oct 9;20(10):e0332137. doi: 10.1371/journal.pone.0332137 (PMC12510515; doi:10.1371/journal.pone.0332137)
Supplement: S1 Appendix — (ZIP) [file pone.0332137.s001.zip › S1 Appendix. 226 original policy documents/36-国家卫生健康委关于贯彻落实《中共中央 国务院关于优化生育政策促进人口长期均衡发展的决定》的通知(FBM-CLI-4-5054640).doc]

国家卫生健康委关于贯彻落实《中共中央 国务院关于优化生育政策促进人口长期均衡发展的决定》的通知

**发布部门：** [国家卫生健康委员会](https://www.pkulaw.com/law/chl?Aggs.IssueDepartment=60321) [机构沿革](javascript:void(0))

**发文字号：**国卫人口发〔2021〕24号

**发布日期：**2021.07.21

**实施日期：**2021.07.21

**时效性：** [现行有效](https://www.pkulaw.com/law/chl?Aggs.TimelinessDic=01)

**效力级别：** [部门工作文件](https://www.pkulaw.com/law/chl?Aggs.EffectivenessDic=XE0303)

**法规类别：** [卫生综合规定](https://www.pkulaw.com/law/chl?Aggs.Category=09601)

国家卫生健康委关于贯彻落实《中共中央 国务院关于优化生育政策促进人口长期均衡发展的决定》的通知
（国卫人口发〔2021〕24号）

各省、自治区、直辖市及新疆生产建设兵团卫生健康委，军队计划生育工作领导小组办公室，中直机关、中央国家机关人口计生委，委机关各司局，委直属和联系单位，中国老龄协会：
　　为深入贯彻落实《中共中央 国务院[关于优化生育政策促进人口长期均衡发展的决定](https://www.pkulaw.com/chl/1247bc81dccaeed3bdfb.html)》（以下简称[《决定》](https://www.pkulaw.com/chl/1247bc81dccaeed3bdfb.html)），现就有关事项通知如下：
　　**一、**深入学习[《决定》](https://www.pkulaw.com/chl/1247bc81dccaeed3bdfb.html)精神
　　[《决定》](https://www.pkulaw.com/chl/1247bc81dccaeed3bdfb.html)是以习近平同志为核心的党中央统筹中华民族伟大复兴战略全局和世界百年未有之大变局，着眼于加快推进现代化建设、实现中华民族伟大复兴作出的一项重大决策。[《决定》](https://www.pkulaw.com/chl/1247bc81dccaeed3bdfb.html)充分肯定计划生育政策的伟大成就和历史贡献，充分肯定党的十八大以来调整完善生育政策取得的显著成效，强调适应人口形势新变化、推动高质量发展新要求，兼顾多重政策目标，实施三孩生育政策及配套支持措施。
　　[《决定》](https://www.pkulaw.com/chl/1247bc81dccaeed3bdfb.html)提出，实施三孩生育政策及配套支持措施具有十分重要的意义，有利于改善人口结构，落实积极应对人口老龄化国家战略；有利于保持人力资源禀赋优势，应对世界百年未有之大变局；有利于平缓总和生育率下降趋势，推动实现适度生育水平；有利于巩固全面建成小康社会成果，促进人与自然和谐共生。
　　[《决定》](https://www.pkulaw.com/chl/1247bc81dccaeed3bdfb.html)指出，坚持以习近平新时代中国特色社会主义思想为指导，坚持以人民为中心、以均衡为主线、以改革为动力、以法治为保障，实施三孩生育政策及配套支持措施，完善服务管理制度，提升优生优育和普惠托育服务水平，降低生育、养育、教育成本，增进家庭和谐幸福，推动实现适度生育水平，促进人口长期均衡发展。

　　**二、**贯彻落实[《决定》](https://www.pkulaw.com/chl/1247bc81dccaeed3bdfb.html)的重点任务
　　（一）依法实施三孩生育政策。要深入评估本地区人口发展形势、工作基础和政策实施风险，科学制定实施方案。及时提请省级人大或其常委会修订本省（区、市）人口与计划生育条例，重点对生育调节、奖励与社会保障、普惠托育服务、计划生育服务和法律责任等相关内容进行修订。取消社会抚养费，清理和废止相关处罚规定，将入户、入学、入职等与个人生育情况全面脱钩。遵循法制统一原则，注重与上位法的协调，做好三孩生育政策实施前后奖励扶助和制约处罚措施的衔接。压实属地责任，依法依规妥善处理历史遗留问题，维护人民群众的合法权益，维护工作大局的稳定。
　　（二）提高优生优育服务水平。以深入实施母婴安全五项制度为主线，启动实施母婴安全行动提升计划。保障儿童健康，实施健康儿童行动提升计划和母乳喂养促进行动，加强儿童医疗保健服务，开展儿童友好医院建设。开展妊娠风险筛查，对孕产妇进行妊娠风险评估分级，严格进行高危专案管理。着力加强各级危重孕产妇和新生儿救治中心建设与管理，组建区域急救专家组，强化转运、用血等重点环节保障，畅通转诊救治绿色通道，提升临床救治能力。健全出生缺陷防治网络，落实三级预防措施。做好出生缺陷患儿基本医疗和康复救助工作。规范人类辅助生殖技术应用，强化规划引领，严格技术审批，加强技术服务监管，保障相关技术规范、有序应用。加快补齐生育相关公共服务短板，落实《“十四五”优质高效医疗卫生服务体系建设实施方案》。加强妇女儿童健康服务能力建设，增加妇产、儿科优质医疗资源供给，实现省、市、县均有1个标准化的妇幼保健机构，努力为群众提供怀得上、孕得优、生得安、育得好的生育全程优质服务。
　　（三）促进普惠托育服务发展。研究编制“十四五”托育服务发展专项规划，明确总体要求、主要目标、重点任务和保障措施，按年度分解重点任务，定期开展跟踪评估，推动各项政策措施落地见效。实施“十四五”公办托育服务能力建设项目和普惠托育服务专项行动，扩大中央预算内投资，支持社会力量发展社区托育服务设施和综合托育服务机构。鼓励有条件的用人单位在工作场所为职工提供托育服务，规范家庭托育服务健康发展，支持隔代照料、家庭互助等照护模式。完善托育行业国家职业技能标准和评价规范，实施从业人员能力提升专项计划。建立健全登记备案、信息公示和质量评估制度，完善综合监管，坚决守住安全和健康的底线。开展全国婴幼儿照护服务示范城市创建活动，推介一批典型经验，培育托育服务民族品牌。
　　（四）保障计划生育家庭权益。对全面两孩政策调整前的独生子女家庭和农村计划生育双女家庭，继续实行现行各项奖励扶助制度和优惠政策。鼓励各地探索设立独生子女父母护理假等制度。健全计划生育特殊家庭全方位帮扶保障制度，动态调整特别扶助制度扶助标准。将特殊家庭老年人作为重点群体，优先安排入住公办养老机构，提供无偿或低收费托养服务，有条件的地方可对生活长期不能自理、经济困难的发放护理补贴。鼓励各地探索建立公益金或基金，重点用于帮扶计划生育特殊家庭。完善政府主导、社会组织参与的扶助关怀工作机制，建立定期巡访制度，积极推进“双岗”联系人制度、就医绿色通道和家庭医生签约等服务，扎牢织密帮扶安全网。深入开展“暖心行动”，为特殊家庭提供生活照料和精神慰藉等服务。
　　（五）持续深化服务管理改革。以“一老一小”为重点，建立覆盖全生命周期的人口服务体系。加强基层服务管理体系和能力建设，增强抚幼养老功能。落实生育登记服务制度，做好生育咨询指导，推行异地网上办理、一站式办理，做好与生育保险等待遇的衔接，最大程度便利群众。会同相关部门，积极推进出生医学证明、户口登记、医保参保、社保卡申领、儿童预防接种等“出生一件事”联办。对人口发展与经济、社会、资源、环境矛盾较为突出的地区，坚持分类指导，因地制宜开展宣传倡导，促进相关惠民政策与生育政策有效衔接，精准做好各项管理服务。改革计划生育优质服务先进单位创建活动，完善创建标准，丰富创建内涵，发挥好示范引领作用。
　　（六）加强人口监测和战略研究。健全基层监测网络队伍，完善人口监测点，推进生育登记、孕产期保健、住院分娩、出生医学证明、儿童预防接种、居民健康档案等信息共享，加强全员人口库建设。完善国家生命登记管理制度，健全覆盖全人群、全生命周期的人口监测体系。加强与教育、公安、人力资源社会保障、民政、统计、医保等部门协作，促进人口服务基础信息融合共享、动态更新。跟踪监测生育形势和人口变动趋势，开展人口形势分析，深化人口与经济社会发展等重大问题研究。加强新时代中国特色人口学科和理论体系建设，发展人口研究高端智库。

　　**三、**切实强化实施保障
　　（一）加强组织领导。将贯彻落实[《决定》](https://www.pkulaw.com/chl/1247bc81dccaeed3bdfb.html)作为卫生健康系统的一件大事，各省（区、市）卫生健康委党组（党委）要组织专题学习培训，深入领会[《决定》](https://www.pkulaw.com/chl/1247bc81dccaeed3bdfb.html)的重大意义和精神实质，明确目标方向和主要任务，切实把思想行动统一到中央决策部署上来。要制定工作清单，明确重点任务和职责分工，主要领导亲自抓，层层传导压力，狠抓任务落实。
　　（二）加强工作协同。积极做好党委政府的参谋助手，完善相关议事协调机制，落实目标管理责任制，主动协调相关部门出台配套支持措施，出实招、办实事、求实效，切实解决群众后顾之忧。充分发挥计生协会等群团组织和各类社会组织在促进人口发展、家庭建设、生育支持等方面的积极作用，形成贯彻落实[《决定》](https://www.pkulaw.com/chl/1247bc81dccaeed3bdfb.html)的合力。
　　（三）加强宣传引导。积极配合宣传部门，充分利用大众传媒，深入宣传计划生育工作的伟大成就，大力宣传优化生育政策的重要意义，弘扬主旋律、汇聚正能量，营造良好舆论氛围。深入开展婚育新风进万家活动，倡导适龄婚育、优生优育，构建新型婚育文化。
　　（四）加强调研指导。我委将深入开展工作调研，进行工作指导。各级卫生健康行政部门要做好自查工作，压实责任，确保[《决定》](https://www.pkulaw.com/chl/1247bc81dccaeed3bdfb.html)落地见效。各省（区、市）卫生健康委要每年向我委报告贯彻落实[《决定》](https://www.pkulaw.com/chl/1247bc81dccaeed3bdfb.html)的情况。

国家卫生健康委
　　2021年7月21日

© 北大法宝：（[www.pkulaw.com](https://www.pkulaw.com/)) 专业提供法律信息、法学知识和法律软件领域各类解决方案。北大法宝为您提供丰富的参考资料，正式引用法规条文时请与标准文本核对。
欢迎查看所有[产品和服务](https://www.pkulaw.com/helps/2.html)。法宝快讯：[如何快速找到您需要的检索结果？](https://www.pkulaw.com/helps/70.html)[法宝](https://www.pkulaw.com/helps/69.html)V6有何新特色？

扫描二维码阅读原文

**原文链接：**<https://www.pkulaw.com/chl/e68dcf51dbfe9de0bdfb.html>
